# Hepatic histopathology and apoptosis in diet-induced-obese mice under *Escherichia coli* pneumonia

**DOI:** 10.18632/aging.101956

**Published:** 2019-05-14

**Authors:** Hetao Song, Zhicai Zuo, Zhuangzhi Yang, Caixia Gao, Kejie Chen, Jing Fang, Hengmin Cui, Ping Ouyang, Junliang Deng, Yi Geng, Hongrui Guo

**Affiliations:** 1 College of Veterinary Medicine, Sichuan Agricultural University, Chengdu 611130, Sichuan, PR China; 2 Chengdu Academy of Agriculture and Forestry Sciences, Chengdu 611130, Sichuan, PR China; 3 School of Public Health, Chengdu Medical College, Chengdu 610500, Sichuan, PR China; * Equal contribution

**Keywords:** *Escherichia coli*-pneumonia, liver, histopathology, apoptosis, obesity

## Abstract

This research was to investigate the difference of hepatic histopathology and apoptosis between the diet-induced obesity (DIO) and normal (lean) mice after *Escherichia coli* (*E. coli*) pneumonia. A total of 128 ICR mice were selected to be challenged intranasally with phosphate-buffered saline (PBS) or 4×10^9^CFUs/mL of *E. coli*, and the liver histopathology and apoptosis were examined pre- and post-infection. Results showed that the liver index, levels of lipid droplets, cytokines, adipocytokines, oxidative stress, apoptotic percentage, and apoptotic related factors in the *E. coli-*infected mice were generally higher than those in the uninfected mice, whereas the hepatic glycogen and Bcl-2 were the opposite. Interestingly, after *E. coli* infection, the DIO-*E. coli* mice exhibited decreased liver index and apoptotic percentages, and reduced levels of TNF-α, IL-6, resistin, MDA, GSH, CAT, Caspase-3, Caspase-9, Bax as well as Bax/Bcl-2 ratio in comparison to the lean-*E. coli* mice. Our results indicated that *E. coli*-induced pneumonia caused hepatic histopathological damage, increased hepatic apoptosis, oxidative damages, and higher levels of cytokines and adipocytokines. However, such changes showed less severely in the DIO mice than in the lean mice following *E. coli* pneumonia.

## INTRODUCTION

Obesity, defined as a state of abnormal or excessive accumulation of adipose tissue due to imbalance in energy consumption and expenditure, increases the risk of various metabolic disease, such as type II diabetes, cardiovascular diseases and steatohepatitis [1]. According to the latest estimation from the World Health Organization, obesity has reached pandemic proportions, and worldwide obesity has nearly tripled since 1975, and more than 650 million adults were obese (body mass index (BMI) ≥ 30kg/m^2^) in 2016 [2]. With disordered metabolism of energy by excesses of adipose tissue, liver, as a center to maintain whole body energy homeostasis [3], is an affected target organ. Obesity plays an important role in the increasing prevalence of non-alcoholic fatty liver disease (NAFLD) [4] and hepatocellular carcinoma in humans [5]. And the obesity induced by high-fat diet is associated with hepatic steatosis and vesicular degeneration in mouse model [6, 7].

Obese individuals, apart from abnormal metabolism, exhibits altered immune state, which is characterized by abnormal production of inflammatory cytokines (e.g tumor necrosis factor (TNF)-α and interleukin (IL)-6 and adipocytokines (e.g resistin and leptin) [8]. And hospitalized obese patients have been shown to be at increased risk for pulmonary aspiration and community-related respiratory tract infections [9, 10]. Of bacteria causing community-acquired pneumonia, *Escherichia coli* (*E. coli*) is the second most common cause, and resulted in mortality of up to 70% reported by Packham and Sorrell [11]. Interestingly, the term “obesity paradox” was coined by Gruberg and colleagues who found that obese patients (BMI ≥ 30kg/m^2^) had lower risk for the in-hospital complications and one-year mortality rates after percutaneous coronary intervention compared with normal-weight (18.5 ≤ BMI ≤ 25 kg/m^2^) [12]. It has confirmed that “obesity paradox” might also be true in the population of obese patients with pneumonia. Nie and colleagues found that based on meta-analysis, obese subjects were significantly associated with reduced risk of pneumonia mortality [13]. The diet-induced obese (DIO) mice, similarly, exhibited less severe lung injury and lower mortality than the lean mice after intranasal instillations of non-fatal dose of *E. coli*, which was associated with the delayed inflammatory response and oxidative stress by diet-induced obesity [14, 15]. Furthermore, the infection-induced lipid metabolic disorders were slighter in the DIO mice than in the lean mice through AMPKα pathway in the state of non-fatal pneumonia caused by instillation *E. coli* [16]*.*

It has been reported that community-acquired pneumonia could induce dysfunction of liver [17], and that lung microvascular permeability, neutrophilic alveolitis, and mortality were augmented if *E. coli* endotoxemia occurred with pre-existing acute liver injury [18], which indicated that liver plays a central role in the response to the *E. coli*-induced pneumonia. To investigate the hepatic histopathology and apoptosis in normal and DIO mice under *E. coli*-induced pneumonia, we performed the present study, in which the histopathology, cytokine and adipocytokine secretion, oxidative stress and apoptosis of liver in the DIO mice and lean mice with non-fatal pneumonia caused by *E. coli* were determined. The data from this research would provide a reference for the further study on the liver injury during bacterial pneumonia between the lean and DIO mice.

## RESULTS

### The body weight, Lee's index and serum triglyceride (TG) and total cholesterol (TC) levels

**Figure 1:**
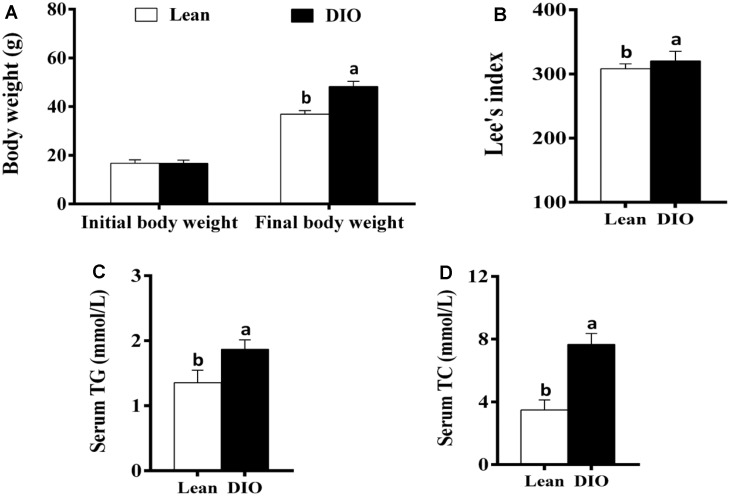
**The changes of body weight, Lee's index and serum TG and TC levels in mice.** (**A**) Body weight; (**B**) Lee's index; (**C**) Serum TG; (**D**) Serum TC. Note: Letter a or b represents difference (*p*<0.05) between the group and lean group or DIO group, respectively.

The initial body weight of mice in the lean and DIO groups showed no significant difference (*p*>0.05). After feeding with the high-fat diets or normal diets for 8 weeks, the final body weight of mice in the DIO group was significantly increased (*p*<0.05) compared with the lean group (Figure 1A). Furthermore, the Lee's index and serum TG and TC levels in the DIO group were significantly higher (*p*<0.05) than those in the lean group (Figure 1B–1D).

### Macroscopic parameters in the liver

**Figure 2:**
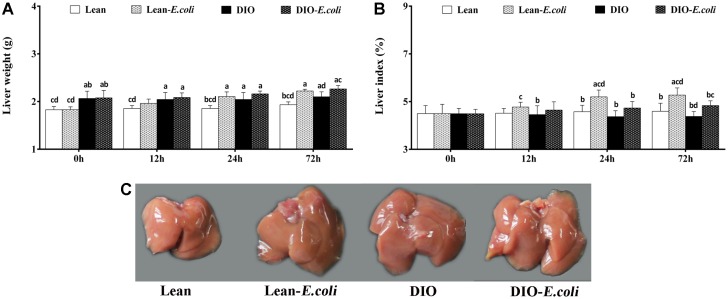
**The changes of liver macroscopic parameters in mice after *E. coli* infection.** (**A**) The liver weight; (**B**) The liver index; (**C**) The liver macroscopic structure at 72h. Note: Letter a, b, c or d represent difference (*p*<0.05) between the group and the lean group, lean-*E. coli* group, DIO group, or DIO-*E. coli* group, respectively.

Before pulmonary *E. coli* infection, the liver weight in the DIO group was higher (*p*<0.05) than that in the lean group, but the liver index showed no significance before infection (*p*>0.05). After *E. coli* infection, compared with the lean group, the liver weight and liver index in the lean-*E. coli* group were significantly increased (*p*<0.05) at 24 and 72h. And the liver weight and liver index in the DIO-*E. coli* group were raised (*p*<0.05) only at 72h in comparison to the DIO group. Moreover, at 24h and 72h post-infection, the liver indexes of the DIO-*E. coli* group were significantly lower (*p*<0.05) than those of the lean-*E. coli* group (Figure 2A and 2B).

Macroscopically, before infected with *E. coli*, lean and DIO mice showed similar hepatic morphological characteristics including color and texture, except that DIO mice exhibited larger hepatic volume than the lean mice. After infected with *E. coli*, liver volumes of the lean- and DIO-*E. coli* mice were larger than those of lean and DIO mice, respectively, especially at 72 h (Figure 2C). This was coincidence with the liver weight showed in Figure 2A.

### Histopathological observation of liver

**Figure 3:**
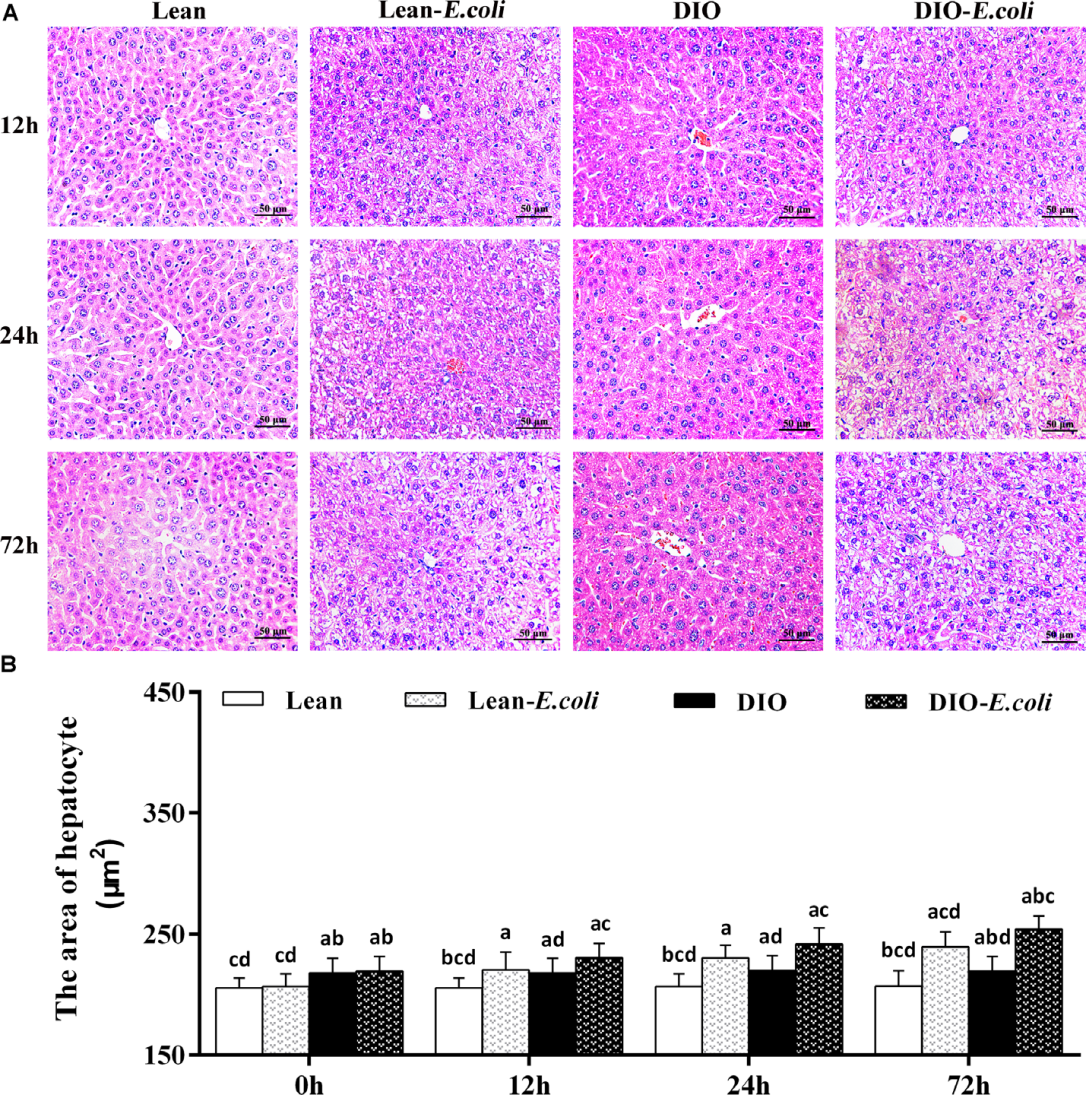
**The histopathological changes of the liver after *E. coli* infection.** (**A**) The representative histopathological images of livers (HE staining, bar=50 μm); (**B**) The area of hepatocyte. Note: Letter a, b, c or d represent difference (p<0.05) between the group and the lean group, lean-E. coli group, DIO group, or DIO-E. coli group, respectively.

Microscopically, in Figure 3A, the histopathological structure of the liver in the lean group was normal with orderly arranged-hepatic cords and obvious hepatic sinusoids, as well as normal hepatocytes. And the histological structure of hepatic tissue in the DIO group was similar to that in the lean group, except for massive fatty droplets in the hepatocytes. After *E. coli* infection at 12h, the hepatic cords were disorganized, and hepatic sinusoids appeared narrow due to the swelling of hepatocytes, and liver steatosis and vesicular degeneration were observed in both the lean- and DIO-*E. coli* groups. Albeit the DIO-*E. coli* group exhibited more serious steatosis and vesicular degeneration than the lean-*E. coli* group from 0h to 72h, the lean-*E. coli* group changed more dramatically. All the pathological changes mentioned above aggravated from 24h to 72h post-infection. As indicated in Table 1, the histological score showed the difference in the severity of steatosis and vesicular degeneration among four groups after infection.

**Table 1:** Histological score of hepatic steatosis and vesicular degeneration.

	**Lean group**	**Lean-*E. coli* group**	**DIO group**	**DIO-*E. coli* group**
**0h**	0	0	2	2
**12h**	0	3	2	4
**24h**	0	4	2	5
**72h**	0	5	2	6

Note: The level of severity was judged from 0 to 6, and the larger the number is, the more serious lesion is.

The area of hepatocyte in the DIO group was larger (*p*<0.05) than that in the lean group before infection. After infection, the area of hepatocyte significantly increased (*p*<0.05) in the lean-*E. coli* group or DIO-*E. coli* group from 12h to 72h compared with the lean group or DIO group, respectively, but it was significantly higher in the DIO-*E. coli* group than in the lean-*E. coli* group at 72h (*p*<0.05) (Figure 3B).

### The integrated optical density of lipid droplets in the liver

**Figure 4:**
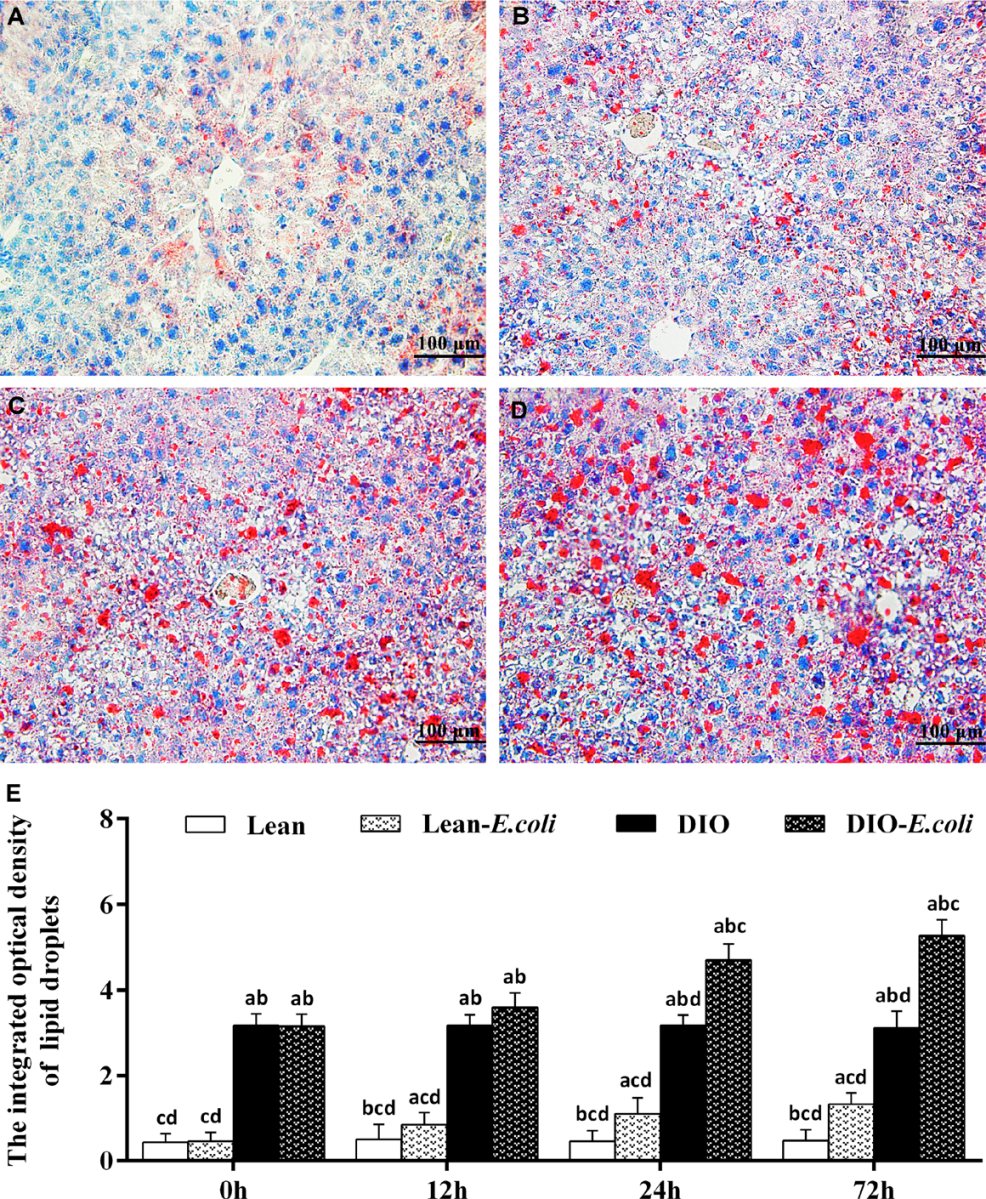
**The changes of lipid droplet deposition in the liver after *E. coli* infection.** (**A–D**) The representative images of the liver lipid droplets deposition at 72h after receiving intranasal instillations (Oil Red O staining, bar=100 μm). (**A**) lean group; (**B**) lean-*E. coli* group; (**C**) DIO group; (**D**) DIO-*E. coli* group; (**E**) the integrated optical density of hepatic lipid droplets. Note: Letter a, b, c or d represent difference (*p*<0.05) between the group and the lean group, lean-*E. coli* group, DIO group, or DIO-*E. coli* group, respectively.

The integrated optical density of lipid droplets was used to evaluate the sum of all lipid droplets in the entire field of view under microscope (Figure 4). Before infection, the integrated optical density was very low in the lean group; but this value was significantly higher in the DIO mice than in the lean mice (*p*<0.05). After *E. coli* infection, from 12h to 72h, the values were significantly elevated (*p*<0.05) in the lean- and DIO-*E. coli* groups in comparison to the lean and DIO groups, respectively. Furthermore, the value in the DIO-*E. coli* group was significantly higher (*p*<0.05) than that in the lean-*E. coli* group from 12h to 72h post-infection, but the increased ratio was higher in the lean-*E. coli* group.

### The integrated optical density of hepatic glycogen

**Figure 5:**
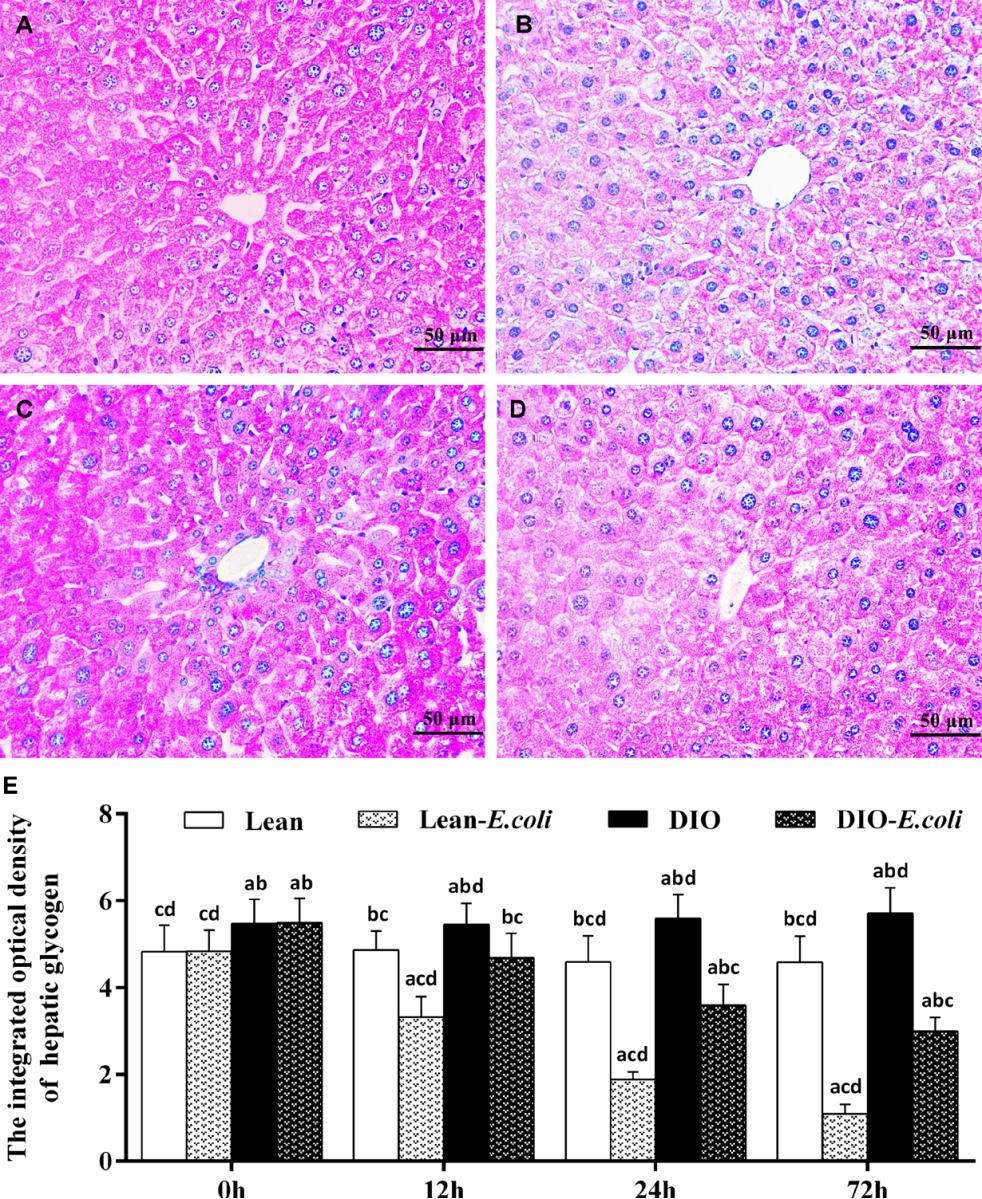
**The changes of hepatic glycogen in the liver after *E. coli* infection.** (**A–D**) The representative images of the hepatic glycogen deposition at 72h after receiving intranasal instillations (PAS staining, bar=50 μm). (**A**) lean group; (**B**) lean-*E. coli* group; (**C**) DIO group; (**D**) DIO-*E. coli* group; (**E**) The integrated optical density of hepatic glycogen. Note: Letter a, b, c or d represent difference (*p*<0.05) between the group and the lean group, lean-*E. coli* group, DIO group, or DIO-*E. coli* group, respectively.

Also, the integrated optical density of glycogen was used to evaluate the sum of glycogen in the entire field of view under microscope (Figure 5). Before infection, the integrated optical density of hepatic glycogen was high both in the lean and DIO groups, and the DIO mice had higher values (*p*<0.05) than the lean mice. After infection, the values decreased (*p*<0.05) both in the lean-*E. coli* group and DIO-*E. coli* group from 12h to 72h, respectively. And the values in the DIO-*E. coli* group were higher (*p*<0.05) than those in the lean-*E. coli* group from 12h to 72h.

### The hepatic apoptosis

**Figure 6:**
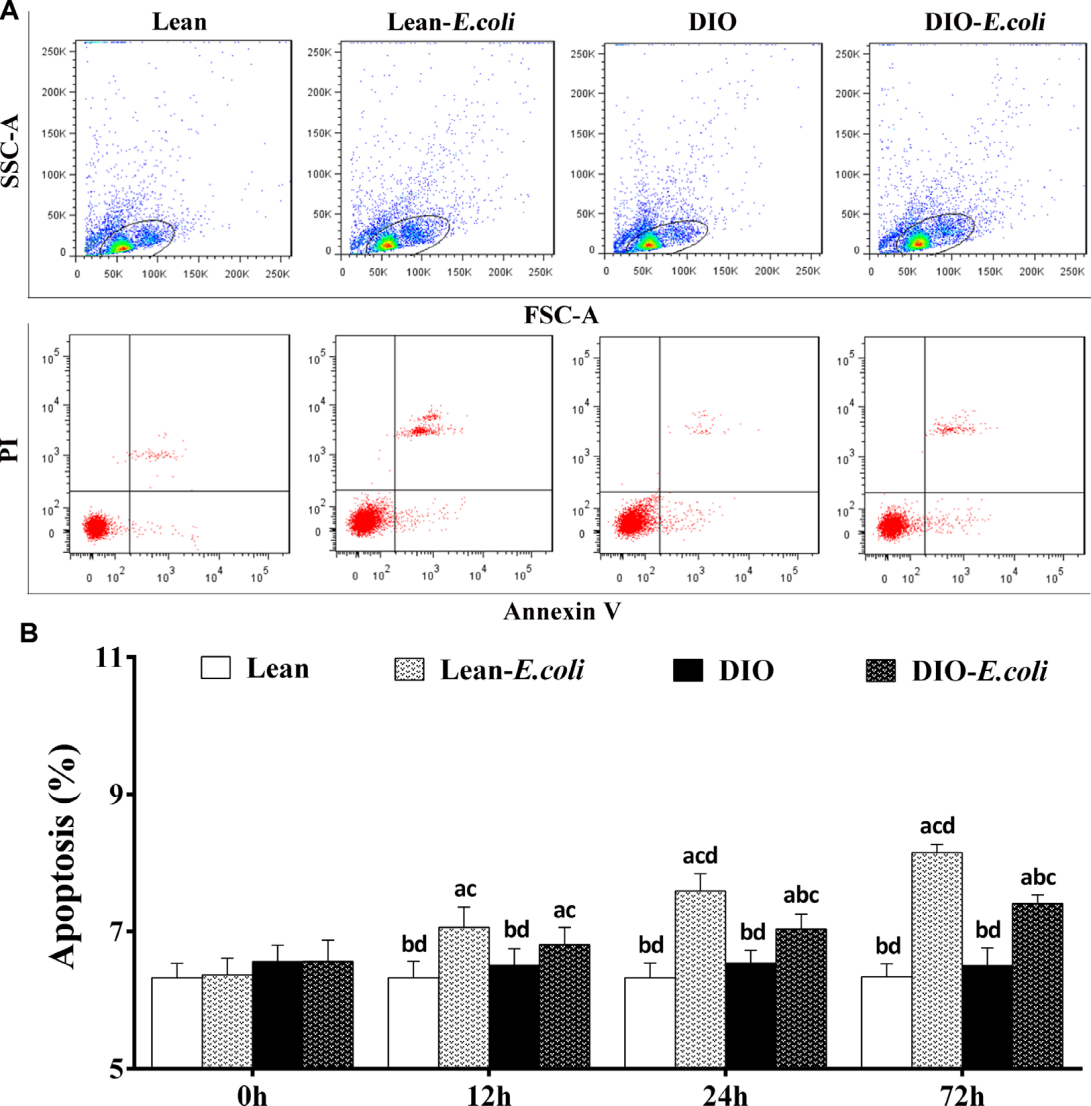
**The percentages of hepatic apoptosis after *E. coli* infection.** (**A**) The representative scatter gram of hepatic apoptosis at 72h after receiving intranasal instillations. (**B**) Hepatic apoptosis percentages. Note: Letter a, b, c or d represent difference (*p*<0.05) between the group and the lean group, lean-*E. coli* group, DIO group, or DIO-*E. coli* group, respectively.

As shown in Figure 6A, cells in the cycle were the objective cells, which means the liver cells including parenchyma cells (hepatocytes) and nonparenchymal cells (like hepatic stellate cell, endotheliocyte and Kupffer's cells). Before being treated with *E. coli*, the percentages of liver apoptotic cells showed no significant difference (*p*>0.05) between the lean group and the DIO group. After being infected with *E. coli*, the values in the lean-*E. coli* group or DIO-*E. coli* group were significantly increased (*p*<0.05) compared with the lean group or DIO group from 12h to 72h, respectively. Moreover, the values in the DIO-*E. coli* group were significantly lower (*p*<0.05) than those in the lean-*E. coli* group at 24h and 72h (Figure 6B).

### The contents of the hepatic cytokines and adipocytokines

**Figure 7:**
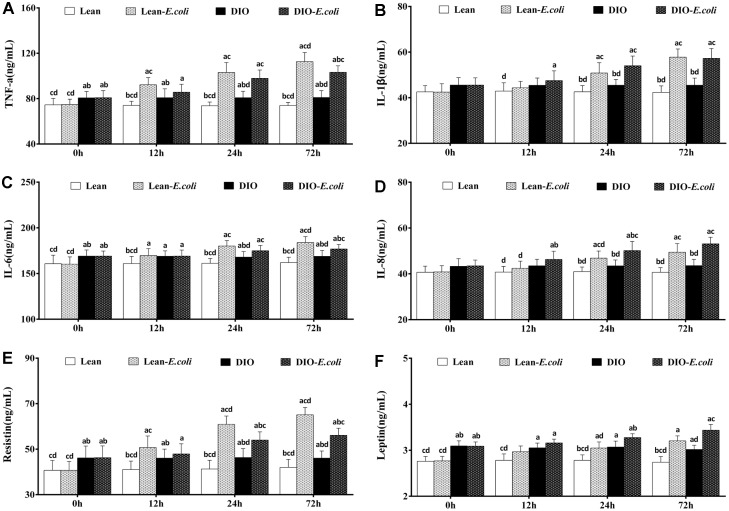
**The contents of cytokines and adipocytokines in the liver after *E. coli* infection.** (**A**) TNF-α; (**B**) IL-1β; (**C**) IL-6; (**D**) IL-8; (**E**) Resistin; (F) Leptin. Note: Letter a, b, c or d represent difference (*p*<0.05) between the group and the lean group, lean-*E. coli* group, DIO group, or DIO-*E. coli* group, respectively.

Before infection with *E. coli*, TNF-α, IL-6, resistin, and leptin contents of the DIO group were significantly higher (*p*<0.05) than those of the lean group. After infection, when compared with the lean group, the contents of TNF-α, IL-1β, IL-6, IL-8, resistin, and leptin in the lean-*E. coli* group were significantly raised (*p*<0.05) from 12h to 72h, except for IL-1β, IL-8, and leptin at 12h. The contents of TNF-α, IL-1β, IL-6, IL-8, and resistin in the DIO-*E. coli* group were dramatically increased (*p*<0.05) at 24h and 72h in comparison to the DIO group, while leptin only at 72h. And the contents of TNF-α, IL-6 and resistin in the DIO-*E. coli* group significantly decreased (*p*<0.05) in comparison to the lean-*E. coli* group at 72h, while the IL-8 increased at 24h (Figure 7).

### The oxidative stress in the liver

**Figure 8:**
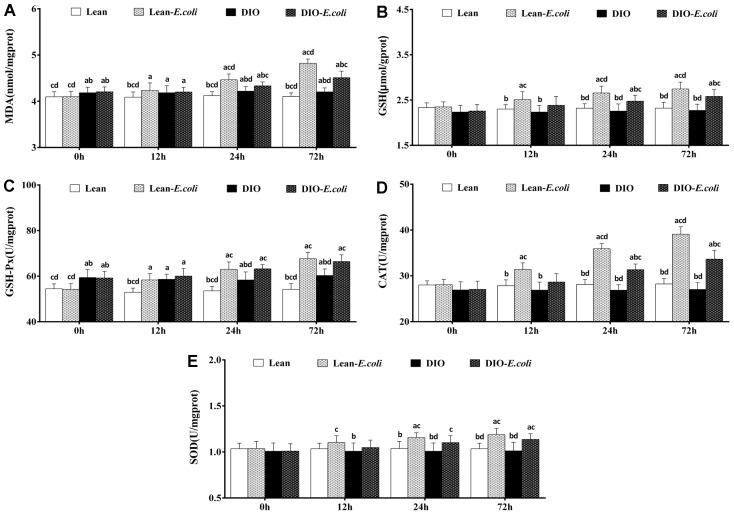
**The changes of oxidative stress in the liver after *E. coli* infection.** (**A**) MDA; (**B**) GSH; (**C**) GSH-Px; (**D**) CAT; (**E**) SOD. Note: Letter a, b, c or d represent difference (*p*<0.05) between the group and the lean group, lean-*E. coli* group, DIO group, or DIO-*E. coli* group, respectively.

As indicated in Figure 8, before infected with *E. coli*, the DIO mice had significantly higher (*p*<0.05) contents of MDA and activity of GSH-Px than the lean mice. From 12h to 72h post-infection, compared with the lean group, the contents of MDA and GSH, and the activities of GSH-Px, CAT and SOD significantly raised (*p*<0.05) in the lean-*E. coli* group, except for SOD at 12h. Meanwhile, the contents of MDA, and GSH and the activities of GSH-Px, CAT, and SOD in the DIO-*E. coli* group significantly elevated (*p*<0.05) at 24h and 72h in comparison to the DIO group. Moreover, compared with the lean-*E. coli* group, the contents of MDA and GSH, and the activity of CAT significantly decreased (*p*<0.05) in the DIO-*E. coli* group at 24h and 72h.

### The expression levels of apoptotic regulatory mRNAs in the liver

**Figure 9:**
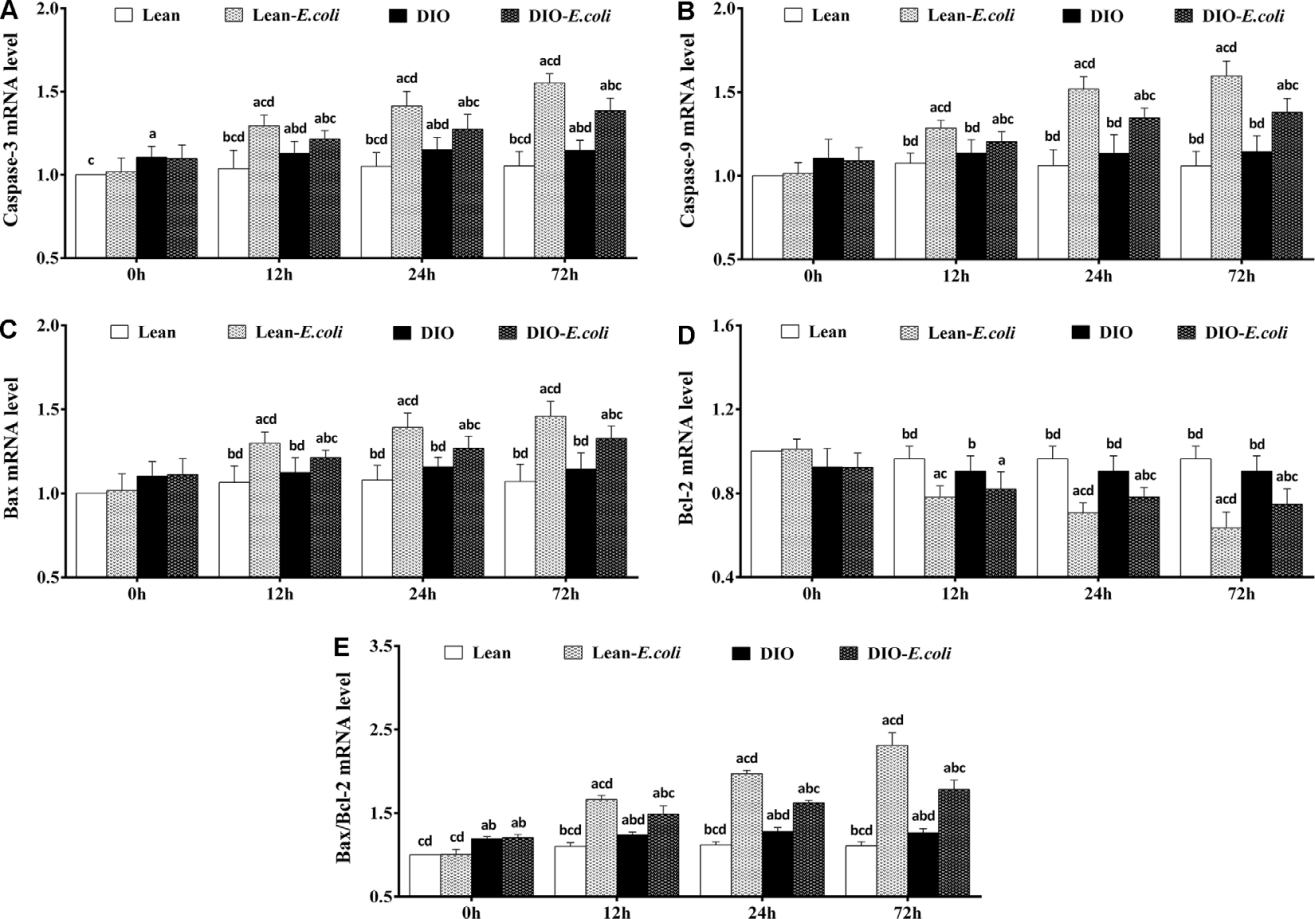
**The changes of mRNA expression levels in the liver (expressed as fold change relative to the lean group) after *E. coli* infection.** (**A**) Caspase-3 ; (**B**) Caspase-9; (**C**) Bax; (**D**) Bcl-2; (**E**) Bax/Bcl-2. Note: Letter a, b, c or d represent difference (*p*<0.05) between the group and the lean group, lean-*E. coli* group, DIO group, or DIO-*E. coli* group, respectively.

The Caspase-3 mRNA and Bax/Bcl-2 mRNA ratio of the DIO group were significantly higher (*p*<0.05) than those of the lean group before infection. From 12h to 72h post-infection, compared with the lean group or the DIO group, the expression levels of Caspase-3, Caspase-9 and Bax mRNA, and Bax/Bcl-2 mRNA ratio in the lean-*E. coli* group or the DIO-*E. coli* group significantly raised (*p*<0.05), while Bcl-2 mRNA significantly decreased (*p*<0.05) in the lean-*E. coli* group (12h to 72h) or the DIO-*E. coli* group (24h and 72h), respectively. Besides, the DIO-*E. coli* group exhibited significantly lower (*p*<0.05) levels of Caspase-3, Caspase-9 and Bax mRNA, and Bax/Bcl-2 mRNA ratio than the lean-*E. coli* group from 12h to 72h, but higher levels of Bcl-2 mRNA at 24h and 72h (Figure 9).

### The levels of apoptotic regulatory proteins in the liver

**Figure 10:**
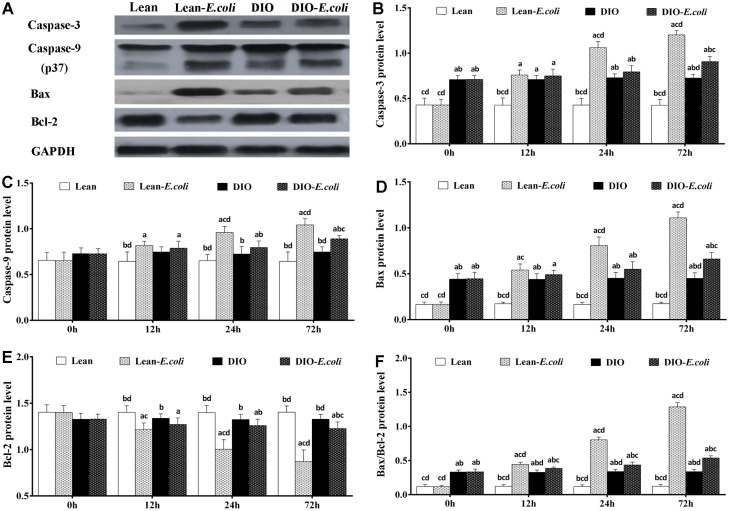
**The changes of protein levels in the mice liver after *E. coli* infection.** (**A**) The representative image of apoptotic regulatory proteins at 72h after receiving intranasal instillations. (**B–F**) Quantitative analysis of the relative protein expression (expressed as fold change relative to the lean group). Note: Letter a, b, c or d represent difference (*p*<0.05) between the group and the lean group, lean-*E. coli* group, DIO group, or DIO-*E. coli* group, respectively.

The protein expression levels of Caspase-3 and Bax, and Bax/Bcl-2 protein ratio in the DIO group were higher (*p*<0.05) than those in the lean group before infection. From 12h to 72h post-infection, the levels of Caspase-3, Caspase-9 and Bax proteins, and Bax/Bcl-2 protein ratio in the lean-*E. coli* group were significantly increased (*p*<0.05), compared with the lean group, while Bcl-2 was significantly decreased (*p*<0.05). Interestingly, Caspase-3, Caspase-9, and Bax proteins in the DIO-*E. coli* group were significantly raised (*p*<0.05) only at 72h, whereas Bcl-2 was decreased (*p*<0.05) only at 72h in comparison to the DIO group. And higher (*p*<0.05) Bax/Bcl-2 protein ratio was observed in the DIO-*E. coli* group compared with the DIO group from 12h to 72h. Compared with the lean-*E. coli* group, moreover, the DIO-*E. coli* group showed significantly decreased (*p*<0.05) levels of Caspase-3, Caspase-9, Bax proteins and Bax/Bcl-2 protein ratio at 24h and 72h, while increased levels of Bcl-2 (Figure 10).

## DISCUSSION

Globally, due to not only obesity *per se*, but obesity-related complications (like type II diabetes, cardiovascular disease and cancer) [19], obesity constitutes a serious threaten to the current and future health of all populations. Recently, Corrales-Medina and colleagues found that obesity may exert a protective effect against 30-day mortality from community-acquired bacterial pneumonia [20]. Our previous studies showed that compared with the lean mice, the diet-induced obese (DIO) mice exhibited lower neutrophil infiltration and contents of proinflammatory cytokines and oxidative stress, and less severe histopathological lesions in the lung, as well as slighter lipid metabolic disorders in the liver after intranasal instillations of non-fatal dose of *E. coli* [14–16]. Moreover, a report has shown that community-acquired pneumonia could induce dysfunction of liver [17]. As known, obese patients have altered physiological metabolism and immune responses. Therefore, in this research, we focused on the liver to investigate the hepatic histopathology and apoptosis in the DIO Mice after *E. coli*-induced pneumonia.

The mice receiving high-fat diet exhibited higher body weight, Lee's index, levels of triglyceride (TG) and total cholesterol (TC) than those receiving normal diet, indicating that the DIO mouse model was established as previously described by Wan et al. [14]. Both the lean and DIO mice showed increased liver weight and liver indexes during the *E. coli* pneumonia, consistent with the larger hepatic volume by macroscopic observation. By paraffin sections and several stains, moreover, the histological structures of liver were obviously influenced by high-fat diet and/or *E. coli* infection. Compared with the lean mice, before *E. coli* infection, the hepatic steatosis and vesicular degeneration were observed in the DIO mice, which may result from the increased level of serum TG. Study in animal models of NAFLD demonstrated that inhibition of TG synthesis resulted in improvement in hepatic steatosis [21]. During the *E. coli* infection, notwithstanding both the lean-*E. coli* and DIO-*E. coli* groups showed hepatic steatosis and vesicular degeneration, such changes in the lean-*E. coli* group were more dramatic than those in the DIO-*E. coli* group, which may contribute to the higher liver index in the lean-*E. coli* group in comparison to the DIO-*E. coli* group after infection. It indicated that the histological structures of liver more drastically worsened in the lean-*E. coli* group caused by *E. coli* infection than by the DIO-*E. coli* group. In addition, high-fat diet increased the reserves of hepatic glycogen, while *E. coli* infection induced the mobilization of liver glycogen in both the lean-*E. coli* group and DIO-*E. coli* group, of which the lean-*E. coli* group was more. Glycogen, as one of two forms of long-term energy reserves, can be synthesized by several enzymes, like glycogen synthase, when the blood glucose rises, otherwise it is decomposed. Jones et al. found that glycogen stores might be consumed by colonized *E. coli* as the source of carbon and energy [22]. In the present study, we found that the reduction of hepatic glycogen in the lean-*E. coli* group was more than that in the DIO-*E. coli* group, which may support the long-term survival of *E. coli* *in vivo* and contribute to the larger *E. coli* loads in the lung of lean mice with *E. coli* pneumonia reported by our previous study [14]. These results indicated that the liver could be not only impacted by *E. coli* pneumonia, but involved in the anti-bacteria of host.

Increase of hepatocyte apoptosis is typically present in patients with NAFLD and in experimental models of steatohepatitis [23]. With hepatic lipid dysregulation caused by *E. coli* pneumonia in the present study, we hypothesized, therefore, that *E. coli* infection caused hepatic apoptosis and impacted differently to the lean- and DIO-*E. coli* groups. Through flow cytometry method, high-fat diet caused increased trend on the hepatic apoptosis in the mice, and *E. coli* infection increased hepatic apoptosis both in the lean- and DIO-*E. coli* groups, in which the lean-*E. coli* group exhibited higher apoptotic percentage of liver than the DIO-*E. coli* group. Apoptosis is a form of programmed cell death that occurs in multicellular organisms, and excessive apoptosis causes atrophy and impairment of organs. Liu et al. revealed that the massive apoptosis of hepatocytes indicated an important step in the development of acute liver injury induced by lipopolysaccharide (LPS) [24]. The data demonstrated that, during the *E. coli*-caused pneumonia, hepatic impairment induced by excessed apoptosis may be more severe in the lean-*E. coli* group than in the DIO-*E. coli* group.

The initiation of apoptosis is tightly regulated by activation mechanisms, and inflammatory mediators and oxidative stress have been confirmed to involved in the process of apoptosis [25]. The hepatic apoptosis is accelerated by several inflammatory mediators, like TNF-α [24]. Conversely, it is inhibited by inflammatory cytokines, such as IL-1β [26], IL-6 [27, 28] and IL-8 [29]. Fukunishi et al. demonstrated that LPS, derived from *E. coli*, can accelerate inflammation via hepatic production of pro-inflammatory cytokines such as TNF-α, and IL-6 [30]. During *E. coli* pneumonia, *E. coli*-derived LPS may reach the liver from the lung by circulation and induce the production of inflammatory cytokines. Moreover, certain adipocytokines, including leptin and resistin, have been reported to act as proinflammatory cytokines [31, 32] and be potential to regulate the process of apoptosis. The leptin exerted anti-apoptotic effect on hepatocytes during acute liver injury in rats [33], as well as resistin [34]. In the present study, the high-fat diet increased the levels of TNF-α, IL-6, resistin and leptin, demonstrating that the pro-apoptotic and anti-apoptotic properties were enhanced. Compared with the DIO-*E. coli* group, the TNF-α contents of the lean-*E. coli* group were increased, which may explain the elevated apoptotic percentage of the liver. However, among other cytokines and adipocytokines exerting anti-apoptosis, the levels of IL-8 and leptin in the DIO-*E. coli* group were higher than those in the lean-*E. coli* group, while the changes of IL-6 and resistin in the DIO-*E. coli* group were lower, which may be associated with the contents of TC. Amundson et al. revealed that obese individuals with high serum TC levels can neutralize circulating LPS, thus decreasing inflammation and inflammatory cytokines [35]. Thereafter, the cytokines or adipocytokines associated with the process of apoptosis could partially explain the increased apoptotic percentage in the liver of the lean-*E. coli* group in comparison to the DIO-*E. coli* group.

Oxidative stress, an imbalance between *in vivo* oxidative and anti-oxidative effects, was estimated by the contents of MDA and GSH, and the activities of GSH-Px, CAT and SOD. MDA is a marker for oxidative stress and the end product of lipid oxidation [36]. GSH is an important antioxidant, which is capable of preventing damage from reactive oxygen species (ROS) under the catalysis of GSH-Px [37]. SOD and CAT, as antioxidases, play critical roles in the elimination of ROS [38]. As is known, obesity is connected with an increase in oxidative stress and formation of ROS [39]. Thus, before *E. coli* infection, the DIO mice exhibited higher MDA content and GSH-Px activity than the lean mice, in line with previous study [40]. Following *E. coli* infection, the contents of MDA and GSH, and the activities of GSH-Px, CAT and SOD significantly increased in the liver of both the lean- and DIO-*E. coli* groups, which demonstrated that *E. coli* infection caused hepatic oxidative stress. The source of the oxidative stress in the liver may result from the inflammation in the lung. It has been confirmed that the phagocytosis of gram-negative bacteria (e.g., *E. coli*) can activate the primary host defense mechanism and result in the generation and release of ROS [41]. The other source may be the LPS produced by *E. coli*, because LPS could increase lipid peroxidation, an index of oxidative stress [42]. After *E. coli* infection, moreover, the contents of MDA, and the activities of GSH and CAT were markedly higher in the lean-*E. coli* group than in the DIO-*E. coli* group. According to the results above, *E. coli* pneumonia caused oxidative stress in the liver of lean- and DIO-*E. coli* groups, and it was more serious in the lean mice.

Oxidative stress is tightly related to the apoptosis. It is well established that mitochondria is the main site of the generation of ROS [26], and, by formation of membrane pores or increasing permeability of the membrane, oxidative stress impairs mitochondria and causes apoptotic effectors to leak out [43], in which the Bcl-2 and Bax are indispensable. Bax permeabilizes the outer membrane of mitochondria rendering apoptotic cascade, which could be counteracted by Bcl-2 [44], and the Bax/Bcl-2 ratio is in positive correlation with apoptosis [45]. Once apoptotic effectors (e.g. cytochrome c) are released from the mitochondria, the apoptosome is therefore formed in the cytoplasm, and actives Caspase-9, which in turn activates Caspase-3. In the present study, through qRT-PCR and Western blot, high-fat diet increased the levels of Caspase-3 mRNA and protein, and Bax/Bcl-2 ratio in the liver, indicating that the hepatic apoptosis was initiated. However, the apoptotic percentage in the liver of the DIO mice was not significantly higher than that of the lean mice, which may result from the anti-apoptotic capacities of the cytokines and adipocytokines, such as IL-6 and resistin. During *E. coli* pneumonia, the mRNA and protein expression levels of Bax, Caspase-9 and Caspase-3, and Bax/Bcl-2 ratio were all elevated in the liver of both the lean- and DIO-*E. coli* groups, while the Bcl-2 was reduced. Compared with the lean-*E. coli* group, the levels of Bax, Caspase-9 and Caspase-3, and Bax/Bcl-2 ratio in the DIO-*E. coli* group decreased, while Bcl-2 increased, consistent with the increase of hepatic oxidative stress. It was supported that, during *E. coli* pneumonia, the oxidative stress induced the apoptosis in the liver, and the liver of lean mice was more sensitive to the *E. coli* pneumonia than that of DIO mice in this aspect.

In conclusion, *E. coli*-induced pneumonia caused liver damages, such as hepatic steatosis, vesicular degeneration, oxidative stress, increased hepatic apoptosis and higher levels of cytokines and adipocytokines. However, such damages showed less severely in the DIO mice than in the lean mice following *E. coli* pneumonia.

## MATERIALS AND METHODS

All experimental animals, methods and procedures were approved by the institute of Animal Care and the Ethics Committee of Sichuan Agricultural University (Approval No: 2012-024, Chengdu, China).

### Mouse model of diet-induced obesity

128 male 21-day-old ICR mice were housed under specific-pathogen-free raising condition. Based on a previous description [14, 15], after feeding with normal diets or high-fat diets for 8 weeks, all mice were weighed, and their lengths were measured. Mice with obese index greater than 20% and significantly high Lee's index, serum triglyceride (TG) and total cholesterol (TC) contents (A110-1 and A111-1, Nanjing Jiancheng Bioengineering Institute, China) were regarded as obese animals. Then, the mice were divided into two groups (64/group) as lean (normal diet) and DIO (high-fat diet) groups, respectively. Both the mice and normal or high-fat diets were purchased from Dossy Animal Center (Chengdu, China).

$$\begin{array}{l} \text{ Obese index }\\ \text{= }\frac{\text{Individual weight of DIO}-\text{Average weight of Lean}}{\text{Average weight of Lean}}\times 100\text{\%} \end{array}$$

$$\text{Lee’s index}=\frac{\sqrt[{3}]{Body\;weight(g)\times 10^{3}}}{Body\;ength(cm)}\times 100\%$$

### *Escherichia coli* (*E. coli*) induced non-fatal pneumonia model

*E. coli* strains were stored in the Veterinary Medical Laboratory of Sichuan Agricultural University. The *E. coli*-induced non-fatal pneumonia model had been established according to previous reports [14, 15]. Then, the lean and DIO mice were subdivided into four groups (32/group) after being intranasally instilled with 40μL PBS or 4×10^9^CFUs/mL *E. coli*, namely, lean, DIO, lean-*E. coli*, and DIO-*E. coli* groups. Mice were sacrificed at 0h (pre-infection), and 12h, 24h, and 72h (post-infection), and then, serum samples and liver tissues from 8 mice in each group were obtained to perform the following assays.

### Measurement of liver weight and liver index

After necropsy, whole livers were removed aseptically from individual animals, weighed, and photographed. Liver index was calculated by the following formula:

$$\text{Liver index }=\frac{\text{Liver weight }(\text{g})}{\text{Body weight }(\text{g})}\times \text{ 1}00\%$$

### Histopathological examination

The liver tissues were observed and then immediately fixed in 4% paraformaldehyde overnight. Subsequently, the tissues were dehydrated through graded alcohol, paraffin embedded, sectioned at 5μm, and processed for H.E. staining and Periodic Acid-Schiff (PAS) for glycogen. Analysis of intracellular lipid droplets was determined using Oil Red O staining kits (Nanjing Jiancheng Bioengineering Institute, China). The liver histopathology was evaluated through the severity score of steatosis and vesicular degeneration (0 to 6). The area of hepatocyte [46], as well as the integrated optical densities of glycogen and lipid droplets were determined using Image-Pro Plus 5.1 (USA). Briefly, photographs of HE, PAS and Oil Red O staining sections were taken with a digital microscope camera system (Nikon DS-Ri1, Japan), respectively. For each section, 10 fields of 0.064 mm^2^ from each image (corresponding to 10 fields at 400× magnification) were analyzed using computer-assisted image-Pro Plus 5.1 (USA) medical morphological analysis software. Briefly, by selecting “color-chosen target” in the option bar of the morphologic analysis system and marking the detected areas (hepatocyte, glycogen and lipid droplets), all the detected areas in the field were marked in color. Then, “calculating” in the option bar was selected to automatically calculate the area of hepatocyte, and the integrated optical density of glycogen and lipid droplets. Integral optical density is the sum of the response intensities of all selected objects in the entire field of view. Integral optical density = average optical density multiplied by the area of the selected object. The higher the integrated optical density, the greater the overall positive reaction intensity.

### Determination of the cytokines and adipocytokines in the liver by ELISA

After necropsy, the liver tissue was homogenized with normal saline through a cell homogenizer in an ice bath and centrifuged at 3,000 r/min at 4°C for 10min. The supernatant was conserved for future analysis. The concentrations of TNF-α, IL-1β, IL-6 and IL-8, leptin and resistin in the liver were measured with mouse ELISA kits (H052, H002, H007, H008, H174 and H175, Nanjing Jiancheng Bioengineering Institute, China) according to the manufacturer’s instructions, respectively.

### Detection of oxidative stress in the liver

After the mice were euthanized, the liver tissue was homogenized with normal saline through cell homogenizer in an ice bath and centrifuged at 3, 000 r/min at 4°C for 10 min to obtain a clear supernatant. After determining the concentration of total protein in the supernatant of the liver homogenate by BCA protein assay, the glutathione (GSH), malonaldehyde (MDA) contents, and glutathione peroxidase (GSH-Px), superoxide dismutase (SOD) and catalase (CAT) activities in the liver supernatant were measured by biochemical method following the instruction of reagent kits (A006-2, A003-1, A005, A001-3 and A007-1, Nanjing Jiancheng Bioengineering Institute, China).

### Apoptosis detection by flow cytometry

The liver tissues were sampled to determine the percentage of apoptotic cells by flow cytometer, similar to the method reported by Chen et al. [47]. Briefly, the excised livers were immediately ground to form a cell suspension and filtered. The cells were washed twice with the cold PBS and were suspended in PBS at a concentration of 1×10^6^cells/mL. Afterward, 5μL of Annexin V-Fluorescein isothiocyanate (V-FITC) and 5μL propidium iodide (PI) were added into 100μL cell suspension, and incubated at 25°C for 15min in the dark. Four hundred microliters of 1×annexin binding buffer (BD Pharmingen, USA, 559763) were added to the mixture, and then, the apoptotic cells were examined by flow cytometry (BD FACSCalibur, USA) within 1h.

### Quantitative real-time PCR (qRT-PCR)

The liver tissues were immediately stored in liquid nitrogen. Then, the liver samples were homogenized in liquid nitrogen, by crushing with a mortar and pestle, and the powdered tissues were collected into eppendorf tubes and stored at -80°C. Total RNA was extracted from frozen liver powders using RNAiso Plus (9108/9109, Takara, Japan). The mRNA was then reverse transcribed into cDNA using Prim-Script™ RT reagent Kit (RR047A, Takara, Japan). And then, the cDNA was used as a template for qRT-PCR analysis. For qRT-PCR reactions, 10 μL mixtures were made by using SYBR^®^ Premix Ex Taq™ II (DRR820A, Takara), containing 5μL Tli RNaseH Plus, 0.4μL of forward and 0.4μL of reverse primer, 3.4μL RNAase-free water and 0.8μL cDNA. Gene expressions of Bax, Bcl-2, Caspase-3, and Caspase-9 were analyzed, and β-actin was used as an internal control [48]. The qRT-PCR data were analyzed with 2^–ΔΔCT^ calculation method [49]. The genes primers were designed with Primer 5 and provided by Sangon Biotech (Shanghai, China). Primers information is provided in Table 2.

**Table 2:** Sequence of primers used in qRT-PCR.

**Gene**	**Accession number**	**Forward primer**	**Reverse primer**
Caspase­3	NM_009810.3	ACATGGGAGCAAGTCAGTGG	CGTCCACATCCGTACCAGAG
Caspase­9	NM_015733.5	GAGGTGAAGAACGACCTGAC	AGAGGATGACCACCACAAAG
Bax	NM_007527.3	ATGCGTCCACCAAGAAGC	CAGTTGAAGTTGCCATCAGC
Bcl-2	NM_009741.5	AGCCTGAGAGCAACCCAAT	AGCGACGAGAGAAGTCATCC
β-actin	NM_007393	GCTGTGCTATGTTGCTCTAG	CGCTCGTTGCCAATAGTG

### Western blot analysis

The liver tissues were lysed and proteins were extracted with RIPA lysis buffer, and then kept in laemmli buffer. Protein samples were separated by SDS-PAGE (10%-15% gels) and transferred to nitrocellulose filter membranes. Membranes were blocked with 5% fat-free milk for 1h and incubated with primary antibodies overnight at 4°C. The primary antibodies were Bax, Bcl-2, Caspase-3, Caspase-9, and GAPDH (Abcam, ab32503, ab182858, ab184787, ab202068, and Cell signaling technology, 5174). The membranes were then washed with Tris-buffered saline (TBS) containing Tween 20 (TBST), and incubated with the biotin-conjugated secondary antibodies (Cell signaling technology, 7074) for 1h, and washed again with TBST. The blots were visualized by ECL^TM^ Chemiluminescence reagent (Beyotime technology, P0018A) and captured on the X-ray film. Then, the statistical data of protein expression was performed with ImageJ2x software.

### Statistical analysis

All the data were analyzed by SPSS 22.0. All the results were expressed as mean ± standard deviation (SD). The significance of difference was analyzed by the independent samples *t* test between two groups, or by variance analyses (LSD or Dunnett’s T3) among four groups. A value of *p*<0.05 was considered significant.
